# ‘Watkins’ & ‘Watkins2.0’: Smart phone applications (Apps) for gait-assessment in normal pressure hydrocephalus and decompensated long-standing overt ventriculomegaly

**DOI:** 10.1007/s00701-024-06275-9

**Published:** 2024-09-28

**Authors:** Kanza Tariq, Lewis Thorne, Ahmed Toma, Laurence Watkins

**Affiliations:** National Hospital for Neurology and Neurosurgery, Queen Square, London, UK

**Keywords:** Smart phone application, 10 metre walking test, Normal pressure hydrocephalus

## Abstract

**Objective:**

Gait disturbance is one of the features of normal pressure hydrocephalus (NPH) and decompensated long-standing overt ventriculomegaly (LOVA). The timed-up-and-go (TUG) test and the timed-10-m-walking test (10MWT) are frequently used assessments tools for gait and balance disturbances in NPH and LOVA, as well as several other disorders. We aimed to make smart-phone apps which perform both the 10MWT and the TUG-test and record the results for individual patients, thus making it possible for patients to have an objective assessment of their progress. Patients with a suitable smart phone can perform repeat assessments in their home environment, providing a measure of progress for them and for their clinical team.

**Methods:**

10MWT and TUG-test were performed by 50 healthy adults, 67 NPH and 10 LOVA patients, as well as 5 elderly patients as part of falls risk assessment using the Watkins2.0 app. The 10MWT was assessed with timed slow-pace and fast-pace. Statistical analysis used SPSS (version 25.0, IBM) by paired t-test, comparing the healthy and the NPH cohorts. Level of precision of the app as compared to a clinical observer using a stopwatch was evaluated using receiver operating characteristics curve.

**Results:**

As compared to a clinical observer using a stopwatch, in 10MWT the app showed 100% accuracy in the measure of time taken to cover distance in whole seconds, 95% accuracy in the number of steps taken with an error ± 1–3 steps, and 97% accuracy in the measure of total distance covered with error of ± 0.25–0.50 m. The TUG test has 100% accuracy in time taken to complete the test in whole seconds, 97% accuracy in the number of steps with an error of ± 1–2 steps and 87.5% accuracy in the distance covered with error of ± 0.50 m. In the measure of time, the app was found to have equal sensitivity as an observer. In measure of number of steps and distance, the app demonstrated high sensitivity and precision (AUC > 0.9). The app also showed significant level of discrimination between healthy and gait-impaired individuals.

**Conclusion:**

‘Watkins’ and ‘Watkins2.0’ are efficient apps for objective performance of 10MWT and the TUG-test in NPH and LOVA patients and has application in several other pathologies characterised by gait and balance disturbance.

**Supplementary Information:**

The online version contains supplementary material available at 10.1007/s00701-024-06275-9.

## Introduction

Normal pressure hydrocephalus (NPH) and long-standing overt ventriculomegaly (LOVA) represent forms of chronic hydrocephalus of adult life. Both conditions feature ventriculomegaly and characteristic symptomatology including disturbances of gait and balance, urinary incontinence and cognitive decline [11, 17, 22]. Early diagnosis and treatment can ameliorate symptoms or inhibit further deterioration [11, 17, 22]. Gait and balance disturbance is not only the earliest manifested and most frequent feature of NPH but is also the most amenable to treatment [1]. It is therefore regarded as obligatory in the diagnosis of NPH [1, 11]. The timed-10-m-walking test (10MWT) and the timed-up-and-go (TUG) test are frequently used diagnostic and prognostic tools for gait and balance disturbances in NPH and LOVA [11, 16, 26]. 10MWT requires the patient to walk a 10 m distance in a straight line. The test can be performed at normal pace or fast pace and heed is paid to the time taken to cover the distance, number of steps taken and the use of any mobility/balance assistance devices such as walking frames or sticks [11]. TUG test entails having a seated patient stand-up and walk 3 m, turn around 180 degrees, walk back and sit down again. Results are analysed by measuring time to complete test as a reflection of gait-velocity [23]. Several studies, including guidelines from the American College of Rheumatology, have aimed to provide an age-matched reference range of average values for both normal pace and fast pace gait speed in both 10MWT [7, 20] and TUG test [6]. The results not only help discriminate between normal and impaired gait, but also facilitate in identifying the risk of fall, frailty, decline in functional activity and cognitive impairment [20]. Comparison of serial results pre- and post-intervention help clinicians monitor the degree and duration of improvement [16, 26].

Traditionally these tests are performed in a controlled clinical environment under direct supervision of a member of the clinical team. A 2021 study by Kuruvithadam et al. showed that patients demonstrated much slower and dysfunctional gait in home environment as compared to clinical environment, possibly due to psychological effect of performance pressure and the relative lack of obstacles and distractions in clinical settings. Notably, real-world measurements displayed superior discrimination of NPH patients as compared to clinical measurements [17].

Recently, technical advances in mobile computing have made it possible to administer these tests in home environment and to compliment them in the clinic. Kuruvithadam et al. performed 10MWT in the patient’s home environment by attaching wearable inertial measurement units (IMUs) to the wrists, ankles and chest of the patient, and extracted the data using a computerised algorithm [17]. Instrumented TUG test (iTUG), utilizing wearable IMUs, accelerometers and gyroscopes, has been performed by several investigators over the decades studying mobility [5], frailty, balance and risk of falls in the geriatric population [10, 21, 27] or in various pathological conditions such as Parkinson’s disease, stroke, cerebral palsy, movement disorders, multiple sclerosis, spinal cord injury and brain injury [24, 25, 28].

The usage of smart-phones for the performance of iTUG has, to date, been undertaken by 5 investigators, all utilizing the built-in accelerometer and gyroscopes of the smart-phone for clinical assessment in such disorders as frailty syndrome, falls-risk, and gait velocity in NPH [8, 13, 14, 19, 28, 30, 29, 30]. With the exception of Milosevic et al. and Yamada et al., all wearable and smart phone iTUG assessments required the data to be migrated from the device and evaluated by skilled personnel using a computerised algorithm.

In 2013 Milosevic et al. developed the first automated smart phone application (app) capable of performing self-administrable TUG tests, aimed at mobility examining in Parkinson’s disease [19]. The app was commercially available on Android systems as smartphone-TUG (sTUG) between 2013 to 2016.

Yamada et al. in 2017 designed a self-administrable automated smart-phone app ‘Hacaro-iTUG’ using iPhone’s built-in accelerometer and gyroscope for gait evaluation in NPH [30]. The test requires for the iPhone to be mounted completely still on the umbilicus with a special belt to prevent any shaking during mobilisation and the placement of a cone at 3 m distance. The results are displayed as a function of time and individuals are assigned a score based on their performance. The scoring system is generated by the developers [30].

In this study we introduce ‘Watkins’ and ‘Watkins2.0’, smart-phone walking apps capable of performing both the 10MWT and the TUG-test, developed for both iOS and Android operating systems, using built-in accelerometers, pedometers and gyroscope. The apps are completed with verbal instructions and hence do not require marking of pre-set distance. It also does not require the smart-phone to be mounted on any specific part of the body. The non-purchase apps calculate and display results as a function of distance covered, time and number of steps taken to cover the distance. To the best of our knowledge these are the first smart-phone apps to include these features and mark the next generation in gait-assessment apps.

## Methodology

### ‘Watkins’ & ‘Watkins2.0’ development

There were two separate applications created for both Android and iOS users. To achieve better accuracy, both the applications were written in native languages for smooth communication of software with hardware, which allowed for data extraction at intervals of 0.005 s. The interaction of hardware with software is described in detail below in their respective sections.

### Android

Android, due to its flexibility and compatibility with almost all devices, has become an essential component of all the hand held devices except Apple devices. There are several platforms available for developers to build applications for android users including react-native, flutter, java, kotlin etc. Out of them, there are few languages that are flexible to both android and iOS and provide utility of cross platform execution. However, when it comes to the usage of hardware, it is difficult to use such languages, and native languages are preferred. In android, the two native languages used to create apps are java and kotlin. The step counter application was written in kotlin. In order to count the steps, several application programming interfaces (APIs) were used to detect the movement of user.

### iOS

iOS possess much higher security and complex structure as compared to Android. However, there are several native platforms to make applications, namely Swift or objective C. Step counter application was written in Swift language. The interface was similar to Android and had the same flow.

### Description

In mobile devices, there are several hardware components that are used for specific movements e.g. gyroscope is used to detect the rotation, accelerometer is used for movement in x,y,z axis and pedometer is used for step-count. Although these hardware components are useful to detect the mobile movement, they have their limitation which prevent them from being used directly. In our application, we used accelerometer, pedometer and gyroscope to detect the linear and angular movement and calculate the number of steps. It is not possible to interact with these hardware components directly. Kotlin provides API calls that interact with hardware following permission coding. Once permitted to access the hardware, we use the APIs to get a response from the hardware. Several open-source libraries were used to get data from the sensors.

An API based call was sent to the hardware that responded by providing coordinates according to the movement of the user. This raw data is then processed and based on the output it is decided whether the user mobilised or not.

For Android this was achieved through libraries such as *android.hardware.SensorEventListener* and *android.hardware.SensorManager*.

For iOS, Core Motion library was used to create events required to communicate with the hardware. One of the main class is the CMMotionManager that manages all the services required to detect the motion. The main hardware used to count steps was pedometer. CMDeviceMotion class provides the attitude, rotation rate, and acceleration of a device. CMPedometer is the most important part of this application, as this class was used to interact with pedometer to detect the movement of the mobile phone based on which it decides how many steps user has taken. Based on the measurements received, steps and distance are calculated.

### Functioning

The application starts with the interface that asks the user for very basic demographics to maintain individual progress records. Following this, the app allows its user to choose which test to perform; TUG test or timed 10-m test.

### The timed 10-m test

**Fig. 1:**
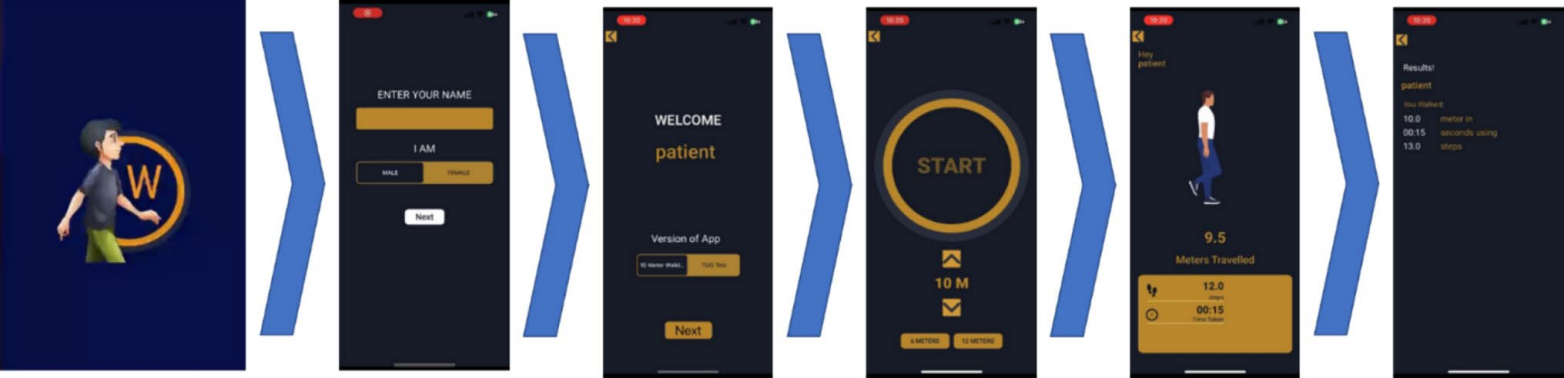
The timed 10-m test in Watkins & Watkins2.0

**Fig. 2:**
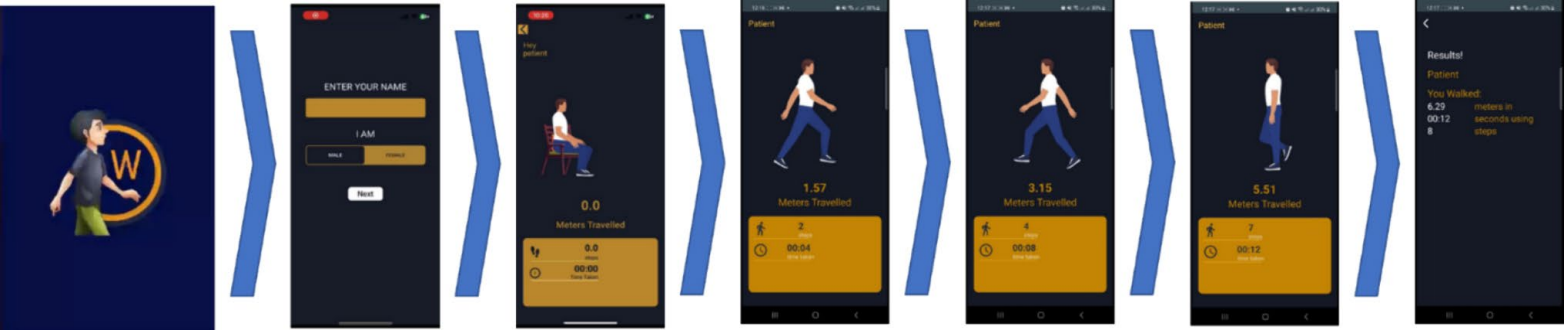
The TUG test in Watkins2.0

The 10MWT is set at default 10 m, but the walking distance can be manually changed. When distance is set, the app gives verbal countdown notice of 3 s before it verbally instructs to ‘start walking’. An animation is displayed that gives information of what the user is doing for example walking forward, backward. When the pre-set distance is covered in a straight line, the app verbally informs the patient to ‘stop’, and displays the result in measure of distance covered, time taken to cover set distance and number of steps taken (Figs. 1 and 2).

### TUG test

The TUG tests is set at default distance of 3 m which can be manually changed. On selecting start, the app verbally countdowns from 3 before asking the user to ‘get up and walk’. Once 3 m are covered the app verbally instructs the user to ‘turn around and walk back to the chair’. Once the required distance is covered the app verbally advises to ‘sit down’ and displays results in terms of time and number of steps taken to cover the distance. The results can be saved and compared with future results.

It is preferred that the app is used in patients not requiring mobility assistance.

### Ethical approval

Formal ethical approval, institutional board review and informed consent from participants was not required prior to the development of the apps. This was confirmed by the Health-Research Authority, UK.

### Validation

The app was validated in 50 healthy adults, 67 NPH and 10 LOVA patients, as well as 5 elderly patients as part of falls risk assessment. 50/67 NPH and 6/10 LOVA patients were invited to use the app in their home-environment and results were compared to walking tests performed under supervision in clinics. The 10MWT was assessed with timed slow-pace and fast-pace. The results were compared with a clinical observer using a stop-watch.

### Statistical analysis

Statistical analysis used SPSS (version 25.0, IBM) by paired t-test, comparing the healthy and the NPH cohorts. Level of precision of the app as compared to a clinical observer using a stopwatch was evaluated using receiver operating characteristics curve.

## Results

### Participant characteristics

The median age of the 50 healthy adults was 48 years (range: 22–56), with 22% (11/50) older than 45, 23 M:27F, 4% (2/50) obese (BMI > 30). The median age of the 67 NPH patients was 77 years (range: 64–89), with 73% (49/67) older than 70, 37 M:30F, 44.7% (30/67) obese (BMI > 30). The median age of the 10 LOVA patients was 47 years (range: 44–56), with 70% (7/10) older than 45, 2 M:8F, 50% (5/10) obese (BMI > 30). The median age of the 5 elderly adults (Parkinson’s disease = 2; dystonia = 2, subdural haematoma = 1) was 62 years (range: 52–74), with 80% (4/5) older than 55, 4 M:1F, none obese. 50 NPH and 6 LOVA patients described the apps as simple, efficient, easy-to-use, eliciting confidence and peace of mind in home settings and highly likely to recommend to others, through patient-reported feedback (Tables 1 and 2).

**Table 1:** Participant characteristics

Participant characteristics	Healthy adults	Normal pressure hydrocephalus	Long-standing overt ventriculomegaly	Elderly adults
Participant number	50	67	10	5
Median age (yrs)	48	77	47	62
M:F	23 M:27F	37 M:30F	2 M:8F	4 M:1F
Percentage obese	4%	44.70%	50%	0%

**Table 2:** Application characteristics

	Accuracy	Error	Sensitivity	Specificity	AUC	p-value
Timed 10-m-walking test						
Time (s)	100%	0.00	100%	100%	1.00	p < 0.001
Number of steps	94.90%	± 1–3 steps	100%	92.60%	0.94	p < 0.001
Distance (m)	97.40%	± 0.25–0.50 m	100%	96.30%	0.978	p < 0.001
**Timed-up-and-go test**						
Time (s)	100%	0.00	100%	100%	1.00	p < 0.001
Number of steps	97.20%	± 1–2 steps	100%	95.60%	0.95	p < 0.001
Distance (m)	87.50%	± 0.50 m	100%	83.30%	0.86	p < 0.001

### Application characteristics

#### Timed -10-m-walking test performed primarily with Watkins

As compared to a clinical observer using a stopwatch the app showed 100% accuracy in the measure of time taken to cover distance (sensitivity 100%, specificity 100%, AUC 1.0), 94.9% accuracy in the number of steps taken with an error ± 1–3 steps (sensitivity 100%, specificity 92.6%, AUC 0.942), and 97.4% accuracy in the measure of total distance covered with error of ± 0.25–0.50 m (sensitivity 100%, specificity 96.3%, AUC 0.978).

#### The timed-up-and-go test performed primarily with Watkins2.0

As compared to a clinical observer using a stopwatch the app has 100% accuracy in time taken to complete the test (sensitivity 100%, specificity 100%, AUC 1.0), 97.2% accuracy in the number of steps with an error of ± 1–2 steps (sensitivity 100%, specificity 95.6%, AUC 0.948), and 87.5% accuracy in the distance covered with error of ± 0.50 m (sensitivity 100%, specificity 83.3%, AUC 0.861).

Significant discrimination in all parameters was seen between healthy and gait-impaired individuals (p < 0.001), with NPH patients having longer duration of time to complete test and a greater number of steps.

## Discussion

### Principal findings

In the current study we present the first self-administrable automated non-purchase gait assessment smart-phone applications capable of performing both 10MWT and TUG test independently with outstanding accuracy, compatible with both Android and iOS systems. Watkins2.0 did, however, have an 87.5% accuracy in measuring the distance covered with error of ± 0.50 m. Similar to previous work conducted by other investigators, the margin of error can be decreased and the percentage of accuracy significantly improved by increasing the total length of distance beyond 3 m [21]. This is due to the subsequent increase in time afforded by the APIs to extract data from the hardware. Both ‘Watkins’ and ‘Watkins2.0’ not only look at the time required to cover a fixed distance but also the number of steps taken to cover the distance. The apps are also capable of calculating the distance covered in meters through the duel input by accelerometer and pedometer. This makes the need to mark a set distance redundant, as the apps, depending on the test being performed, verbally inform the participants to either stop or change direction once the set distance is covered. Unlike other studies, the phone can be held in hand at the time of the assessment and does not need to be fixed on the body [10, 19, 24, 30, 31]. Therefore no additional hardware in the form of special belts or sleeves is required. The results are immediately displayed, as measures of easily understandable parameters such as time, number of steps and distance, instead of complicated scoring systems [30, 31]. This provides ease to the participants in interpreting their results and monitoring their progress and contributes to reassurance. Due to their easy utility and high precision, both apps, specially ‘Watkins’, they were highly recommended to others by every participant who used them in their home. We believe that these are very useful apps and have application even beyond NPH and LOVA.

### Utility and literature review

In recent decades global demographics have changed with a sharp rise in the elderly population [28]. This has also impacted health-care services producing a need to assess health, level of independent function, mobility, balance, cognition and environmental safety in the geriatric population and to identify the vulnerable [28]. Evaluation of functional impediment and performance level of daily activity through measurements of walking speed and assessment of gait pattern are frequently employed tools to predict health status and vulnerability in the elderly [17, 20].

Patients with NPH often feel symptomatic improvement following shunt insertion surgery or spinal-tap test which may last up to six months [1, 16, 26], after which patients may begin to plateau which in some cases causes distress, anxiety and apprehension regarding shunt function and disease prognosis. ‘Watkins’ and ‘Watkins2.0’ allow patients to perform repeat assessments in their home and have an objective estimation of their progress.

These tests have further clinical application in predicting risk of falls in suspected NPH patients [11], helping with early diagnosis of dementia [9], studying frailty in chronic obstructive pulmonary disease [2], monitoring the efficacy of physiotherapy in improving gait in patients with neurological impairment secondary to traumatic brain injury [29] and even in predicting death in the elderly [4]. In 2021 De-Bruijn et al. published a study showing that a majority of the educated population used health apps regardless of age and gender and that they displayed healthier habits [12]. Thus, the app can also be used to help patients improve gait and functional mobility by repeated practice and self-monitoring [18].

### Strengths, limitations and future work

Smart-phone apps are difficult and expensive to make [3], with an ever-increasing amount of technical skill required to develop and improve the app. This, on one hand, results in just a couple of smart-phone apps globally capable of conducting self-administrated clinical assessments accurately and with precision, making ‘Watkins’ and ‘Watkins2.0’ the first of their kind, and on the other hand, greater time will be required to upgrade the apps to improve accuracy in the calculation of distance in the TUG-test and to include gait pattern assessment and supplementary angular measurements of trunk position during different phases of walking. ‘Watkins’ and ‘Watkins2.0’ have the potential to be used as a regular clinical assessment tool in hospitals as well as homes in a variety of neurosurgical, neurological and movement disorders as well as in healthy geriatric population, and future upgrades would aim to include specific features to facilitate assessments in these various conditions.

## Conclusion

**‘**Watkins’ and **‘**Watkins2.0’ represent the next generation in smart-phone apps, capable of objective and efficient performance of 10MWT and the TUG-test in NPH and LOVA patients and have application in several other pathologies featuring gait and balance disturbance.

## Supplementary Information

Below is the link to the electronic supplementary material.

Supplementary file1 (DOCX 15 KB)

## Data Availability

No datasets were generated or analysed during the current study.
