# Supplementary material for: ‘Watkins’ & ‘Watkins2.0’: Smart phone applications (Apps) for gait-assessment in normal pressure hydrocephalus and decompensated long-standing overt ventriculomegaly
Source: Acta Neurochir (Wien). 2024 Sep 28;166(1):386. doi: 10.1007/s00701-024-06275-9 (PMC11436405; doi:10.1007/s00701-024-06275-9)
Supplement: Supplementary file 1 — Supplementary file1 (DOCX 15 KB) [file 701_2024_6275_MOESM1_ESM.docx]

**Supplementary Data**

[**https://github.com/drkanzatariq17/Watkins-App**](https://github.com/drkanzatariq17/Watkins-App)
